# Uncovering the Potential Somatic Angiotensin-Converting Enzyme (sACE) Inhibitory Capacity of Peptides from *Acheta domesticus*: Insights from In Vitro Gastrointestinal Digestion

**DOI:** 10.3390/foods13213462

**Published:** 2024-10-29

**Authors:** Carla S. S. Teixeira, Bruno Carriço-Sá, Caterina Villa, Joana Costa, Isabel Mafra, Isabel M. P. L. V. O. Ferreira, Miguel A. Faria, Tânia G. Tavares

**Affiliations:** 1 REQUIMTE-LAQV, Faculty of Pharmacy, University of Porto, Rua de Jorge Viterbo Ferreira, 228, 4050-313 Porto, Portugal; bfsa@ff.up.pt (B.C.-S.); cvilla@ff.up.pt (C.V.); jbcosta@ff.up.pt (J.C.); isabel.mafra@ff.up.pt (I.M.); isabel.ferreira@ff.up.pt (I.M.P.L.V.O.F.); mfaria@ff.up.pt (M.A.F.); 2 LEPABE—Laboratory for Process Engineering, Environment, Biotechnology and Energy, Faculty of Engineering, University of Porto, Rua Dr. Roberto Frias, 4200-465 Porto, Portugal; 3 ALiCE—Associate Laboratory in Chemical Engineering, Faculty of Engineering, University of Porto, Rua Dr. Roberto Frias, 4200-465 Porto, Portugal

**Keywords:** *Acheta domesticus*, gastrointestinal digestion, bioactive peptides, angiotensin-converting enzyme inhibitory capacity, hypertension

## Abstract

Entomophagy is being proposed as a sustainable and nutritious alternative protein source. Additionally, insect consumption is also associated with some health benefits mediated by bioactive compounds produced during gastrointestinal (GI) digestion. The antihypertensive property resulting from the inhibition of the somatic angiotensin-converting enzyme (sACE) by small peptides is one of the most common bioactivities related to insect consumption. This study aimed to investigate the potential sACE-inhibitory capacity of six peptides (AVQPCF, CAIAW, IIIGW, QIVW, PIVCF, and DVW), previously identified by the in silico GI digestion of *Acheta domesticus* proteins, validate their formation after in vitro GI digestion of *A. domesticus* by LC-MS/MS, and assess the bioactivity of the bioaccessible digesta. The results showed that the IC_50_ values of AVQPCF, PIVCF, and CAIAW on sACE were 3.69 ± 0.25, 4.63 ± 0.16, and 6.55 ± 0.52 μM, respectively. The obtained digesta demonstrated a sACE-inhibitory capacity of 77.1 ± 11.8 µg protein/mL extract (IC_50_). This is the first report of the sACE-inhibitory capacity attributed to whole *A. domesticus* subjected to GI digestion without any pre-treatment or protein concentration. This evidence highlights the potential antihypertensive effect of both the digesta and the identified peptides.

## 1. Introduction

In recent years, the predicted growth of the world’s population, together with the effects of climate change, has forced the world to rethink its sources of feed and food. Meat and animal products are important sources of nutrients, but livestock production is the agricultural/food sector that contributes the most to greenhouse gas emissions [1]. Entomophagy is being proposed as a sustainable and nutritious alternative source of protein compared to traditional meats such as poultry, beef, or pork [2]. Rearing insects have several environmental and economic advantages compared to other animals including lower greenhouse gas and ammonia emissions, a high reproduction rate, high feed conversion efficiency, less need for farmland, efficient use of water and soil, and the need for less economic investment in technology [3,4,5]. Insects are also an excellent source of essential amino acids, monounsaturated fatty acids, fiber, minerals, and vitamins. Their amino acid composition is similar to that of conventional meat, but they contain a higher proportion of crude protein (40 to 75% on a dry weight basis) [2]. The consumption of insects is also associated with some health benefits that have been recognized by traditional medicine for centuries, although there is little scientific evidence to support this [6]. In recent years, several studies have shed light on this issue, identifying some of the compounds that mediate these claimed effects and elucidating their molecular mechanisms. Some of these bioactive compounds are small peptides of 2 to 20 amino acids derived from the gastrointestinal (GI) digestion of insect proteins. Recently, 212 potentially bioactive peptides with antihypertensive, antioxidant, antidiabetic, anti-inflammatory, anti-obesity, hypocholesterolemic, antimicrobial, antithrombotic, anti-Severe Acute Respiratory Syndrome Coronavirus type 2 (SARS-CoV-2), and immunomodulatory properties were registered from the hydrolysates of 13 species of edible insects [7].

The antihypertensive property is one of the most widespread bioactivities associated with insects. According to the World Health Organization [8], hypertension is a leading cause of premature death worldwide, affecting 1.28 billion adults between the ages of 30 and 79. This bioactivity is mediated by the inhibition of the somatic angiotensin-converting enzyme (sACE) (EC 3.4.15.1), a zinc-containing metalloproteinase that is mainly expressed in the lung, but it can also be found in other tissues (e.g., brain, heart, small intestine, ileum, jejunum, and duodenum) with lower expression in the kidney and liver [9]. It has a wide range of substrates and activities, but the most relevant is the cleavage of decapeptide angiotensin-I (Ang-I) into the vasopressor octapeptide angiotensin-II (Ang-II) from the renin-angiotensin system and the degradation of the vasodilator bradykinin from the kallikrein-kinin system through its dipeptidyl carboxypeptidase activity. This justifies the extensive use of sACE inhibitors in the treatment of hypertension and cardiovascular diseases. The vasoconstrictor Ang-II acts on the angiotensin type 1 (AT1) receptor to constrict blood vessels, increase blood pressure, stimulate aldosterone secretion, and produce positive inotropic and chronotropic effects on the heart [10]. The vasodilator bradykinin can release endothelial cytokines, such as nitric oxide (NO) and prostaglandins, to relax blood vessels and inhibit the proliferation of vascular smooth muscle cells. The sACE is responsible for maintaining the equilibrium between the vasoconstrictive and salt-retaining properties of Ang-II as well as the vasodilatory and natriuretic properties of bradykinin [11]. The function of sACE inhibitors is to alter this balance by decreasing the formation of angiotensin II and the degradation of bradykinin. This effectively lowers the mean arterial blood pressure as well as the systolic and diastolic blood pressures in both hypertensive and normotensive subjects.

*Acheta domesticus* [12], commonly known as house cricket, is the third of four EU-regulated insect species for human consumption (*Tenebrio molitor* [13,14], *Locusta migratoria* [15], and *Alphitobius diaperinus* [16]). It is easy to farm and produces nutritious flour with a better taste and flavor profile compared to other edible crickets [17]. In addition to its high nutritive value, *A. domesticus* extracts have been associated with relevant antioxidant and antilipidemic properties [18,19]. In a recent study, Teixeira et al. [20] employed an in silico approach to investigate the potential presence of bioactive peptides derived from the simulated GI digestion of proteins from *A. domesticus* that exhibited a potential inhibitory capacity against the sACE, and consequently, an antihypertensive effect. The peptides AVQPCF, CAIAW, IIIGW, DATW, QIVW, PIVCF, and DVW were identified for the first time as potential inhibitors of sACE, thereby acting as potential nutraceutical or therapeutic agents against hypertension. Although in silico studies are efficient tools for prospecting bioactive peptides with economic and environmental benefits [20,21,22,23,24,25,26], they require experimental validation. Therefore, this study aimed to investigate the potential sACE-inhibitory capacity of six peptides previously identified with the in silico GI digestion of *A. domesticus* proteins, validate their formation after in vitro GI digestion, and further investigate the bioactivity of the bioaccessible fraction of digesta.

## 2. Materials and Methods

### 2.1. Materials and Reagents

The peptides (purity ≥ 98%) were synthesized by GenScript Biotech Corporation (Piscataway, NJ, USA). The dried *A. domesticus* insects were supplied by Portugal Bugs (SFP—Sustainable Food Products Lda, Esposende, Portugal).

The rabbit lung, gastric pepsin, bile salts, pancreatin, phenyl methane sulfonyl fluoride (PMSF), and bovine serum albumin (BSA) were from Sigma-Aldrich (St. Louis, MO, USA). o-Abz-Gly-p-Phe(NO_2_)-Pro-OH was from Bachem (Bubendorf, Switzerland). Dimethyl sulfoxide (DMSO) was purchased from Duchefa Biochemie (Haarlem, The Netherlands). Tris-base buffer, zinc chloride (ZnCl_2_), sodium chloride (NaCl), calcium chloride (CaCl_2_), calcium chloride dihydrate (CaCl_2_(H_2_O)_2_), sodium hydroxide (NaHO), and hydrogen chloride were purchased from VWR (Radnor, PA, USA). Acetonitrile (ACN) and formic acid (FA) of high-performance liquid chromatography (HPLC) grade were obtained from Merck (Darmstadt, Germany). Ultrapure water, purified with a “Seral” system (SeralPur Pro 90 CN), was used. Total peptide quantification was performed via the Pierce^TM^ quantitative colorimetric peptide assay (Pierce BCA Protein Assay Kit, Thermo Fisher Scientific, Waltham, MA, USA).

### 2.2. Preparation of Pure Peptides to Assess sACE-Inhibitory Capacity

From the in silico analysis, seven peptides were identified as potential inhibitors of sACE. However, after several attempts, the synthesis of the peptide DATW was impossible by the company. Therefore, the study proceeded with the remaining six peptides. To ensure efficient solubilization, the pure peptides AVQPCF, QIVW, CAIAW, PIVCF, IIIGW, and DVW (1.0 mg) were first solubilized in 50 μL of DMSO, completing with 950 μL of ultrapure water for a final concentration of 1 mg/mL, following the manufacturers’ indications for the synthesized peptides.

### 2.3. sACE-Inhibition Assays

The sACE-inhibitory capacity was determined using a fluorimetric assay, as reported by Tavares et al. [27], with some modifications. Briefly, the ACE working solution was prepared according to Murray et al. [28] using rabbit lung posteriorly diluted in 0.15 M Tris-base buffer (pH 8.3) with 0.1 mM ZnCl_2_. A total of 40 µL of the working solution was added to each 96-well microplate well (Porvair, Norfolk, UK), then adjusted to 80 µL by adding distilled water to the blank (B), control (C), or samples (S). The enzyme reaction was started by adding 160 µL of 0.45 mM o-Abz-Gly-p-Phe(NO_2_)-Pro-OH dissolved in 0.15 M Tris-base buffer (pH 8.3) containing 1.125 M NaCl, and the mixture was incubated at 37 °C for 30 min. The fluorescence generated was measured using a multiscan microplate fluorimeter (FLUOstar optima, BMG Labtech, Ortenberg, Germany) with excitation and emission wavelengths of 350 and 420 nm, respectively. Data were processed with the software FLUOstar control (version 1.32 R2, BMG Labtech, Ortenberg, Germany). The assay was performed in triplicate, and the resulting data were subjected to a nonlinear fit using GraphPad Prism 5 to determine the IC_50_ (half maximal inhibitory concentration) values. The resulting data (*n* = 3) were expressed as the mean ± SD (standard deviation).

### 2.4. In Vitro GI Digestion

The dried *A. domesticus* insects were ground and submitted to the standardized in vitro GI digestion protocol INFOGEST 2.0 [18], which simulates the oral, gastric, and intestinal phases. Digestions were performed in quadruplicate. The simulated salivary (SSF), gastric (SGF), and intestinal (SIF) fluids were prepared according to Table S1 (Supplementary Materials). To avoid precipitation, CaCl_2_(H_2_O)_2_ was added simultaneously with the fluids during the digestion protocol. The GI protocol was performed in an orbital shaker–incubator (ES-20, BioSan, Riga, Latvia) with an integrated horizontal shaker at 170 rpm and 37 °C.

The oral phase was simulated by the sequential addition of 800 µL of SSF, 195 µL of ultrapure H_2_O, and 5 µL of 0.3 M CaCl_2_(H_2_O)_2_ to 1 g of ground *A. domesticus* (1:1 *w*/*v*), which corresponded to approximately 12 crickets. Mashing was simulated using a glass rod for 30 s, and the mixture was incubated for 2 min. Afterward, gastric digestion was simulated by mixing the masticated mixture with 1600 µL of SGF (1:1 *w*/*v*), 1 µL of 0.3 M CaCl_2_(H_2_O)_2_, and gastric pepsin (2000 U/mL). The pH was adjusted to 3.0 with 6 M HCl, and a final volume of 4 mL was completed with ultrapure water. Mixtures were then incubated for 2 h at 37 °C. The intestinal phase was simulated by incorporating 1700 µL of SIF (1:1 *w*/*v*), 8 µL of 0.3 M CaCl_2_, 10 mM of bile salts, and pancreatin (100 U/mL). The pH was adjusted to 7.0 with 1 M NaOH and the final volume of 8 mL was completed with ultrapure water. The enzymatic activity was stopped by adding 0.1 M of phenyl methane sulfonyl fluoride (PMSF). All tubes were centrifuged at 9000× *g* for 15 min (4 °C), and the supernatant was collected. Two *A. domesticus* replicate digests (AdD1 and AdD2) were lyophilized and used for LC-MS/MS analysis, while the other two were frozen and stored at −20 °C for use in the sACE capacity assays.

### 2.5. Total Peptide Quantification of A. domesticus Digesta

The total peptide quantification of the *A. domesticus* digesta was performed via the Pierce^TM^ quantitative colorimetric peptide assay (480 nm). The calibration curve was generated using BSA as the standard (0 to 500 μg/mL). The analysis used a CLARIOstar^®^ High-Performance Monochromator Multimode from BMG Labtech (Ortenberg, Germany). Each calibration curve standard and the samples were measured in duplicate.

### 2.6. Preparation of Pure Peptides and Digesta for LC-MS/MS Analysis

The pure peptides AVQPCF, QIVW, CAIAW, PIVCF, IIIGW, and DVW were first dissolved in 1 mL of 3% (*v*/*v*) ACN containing 0.1% (*v*/*v*) aqueous FA. A working solution was prepared by combining appropriate volumes of each peptide. This resulted in concentrations of 250 fmol/μL for DVW and 50 fmol/μL each for AVQPCF, QIVW, CAIAW, PIVCF, and IIIGW. All pure peptide solutions were then stored at −80 °C until analysis.

All replicate digests (AdD1.1, AdD1.2, AdD2.1, and AdD2.2) were diluted to a concentration of 350 ng/μL using 3% (*v*/*v*) ACN containing 0.1% (*v*/*v*) aqueous FA.

### 2.7. LC-MS/MS Data Acquisition

The peptide analysis was conducted using an EASY nLC II system coupled to an Impact HD from Bruker Daltonics (Billerica, MA, USA). This configuration featured a CaptiveSpray nanoBooster utilizing ACN as the dopant, as previously detailed [29,30]. The ionization source parameters were set as follows: a capillary voltage of 1300 V, nanoBooster pressure at 0.2 Bar, dry gas flow rate at 3.5 L/min, and a temperature of 150 °C. For pure peptides and sample digesta analysis, 1 μL of the solution was loaded onto an EASY-nLC II system. This was equipped with a μPAC™ Trapping Column from PharmaFluidics (Zwijnaarde, Belgium). The samples were then desalted at a flow rate of 10 μL/min using 0.1% (*v*/*v*) aqueous FA for 4 min. Subsequent peptide separation was achieved on an analytical column RP-C18 μPAC^TM^ (PharmaFluidics, Zwijnaarde, Belgium), 50 cm in length, with a pillar array backbone at an interpillar distance of 2.5 µm. Chromatographic separation was performed using a mixture of two eluents: a mobile phase A consisting of 0.1% (*v*/*v*) aqueous FA, and a mobile phase B composed of 90% (*v*/*v*) ACN with the addition of 0.1% (*v*/*v*) aqueous FA. Elution was performed at a flow rate of 500 nL/min, and the chromatographic separation was accomplished as follows: 0 min (A, 100%), 1 min (A, 98%), 2 min (A, 95%), 61 min (A, 65%), 66 min (A, 10%), and 86 min (A, 10%).

MS acquisition was performed using the middle-band multiple reaction monitoring (MRM) method for the ions [M + H]^+^ of DVW (419.1925 *m*/*z*), QIVW (545.3082 *m*/*z*), CAIAW (563.2646 *m*/*z*), PIVCF (578.3007 *m*/*z*), IIIGW (601.3708 *m*/*z*), and AVQPCF (664.3123 *m*/*z*). All spectra were acquired in positive polarity in the range 150–2200 *m*/*z*.

### 2.8. Statistical Analyses

Experiments were performed with three independent assays. Statistical analysis was performed with SPSS 27.0 (SPSS Inc., Chicago, IL, USA). A multifactorial analysis of variance (ANOVA) was used to compare the ACE-inhibitory capacity of peptides, followed by the Tukey test for multiple data comparisons. Differences were accepted as statistically significant at *p* < 0.05.

## 3. Results and Discussion

A previous study of Teixeira et al. [31] performed the in silico GI digestion of *A. domesticus* proteins, namely Acyl-CoA Delta12-desaturase, diuretic hormone receptor, and Acyl-CoA Delta-9 desaturase, generating seven peptides (AVQPCF, CAIAW, IIIGW, DATW, QIVW, PIVCF, and DVW) with potential antihypertensive properties due to their ability to inhibit the sACE. This result suggests that the consumption of whole *A. domesticus* could have the potential to originate peptides with antihypertensive properties. To validate this hypothesis, the results of the sACE-inhibitory capacity of the generated peptides and of the whole dried *A. domesticus* subjected to in vitro GI digestion were analyzed in detail.

### 3.1. Assessment of the sACE-Inhibitory Capacity of Pure Peptides

Only six out of the seven peptides previously identified [31] as potential sACE inhibitors were synthesized because DATW could not be produced. The sACE-inhibitory capacity of the pure peptides was determined to validate their predicted potential. The results are presented in Table 1.

The peptides exhibiting the best results were AVQPCF (3.69 ± 0.25 µM), PIVCF (4.63 ± 0.16 µM), and CAIAW (6.55 ± 0.52 µM), with IC_50_ values below 10 µM, followed by IIIGW with a value of 54.5 ± 12.6 µM and QIVW and DVW with values of 140.1 ± 10.4 and 195.5 ± 47.4 µM, respectively.

The comparison of the IC_50_ values obtained for the aforementioned peptides with other literature reports is summarized in Table S2 (Supplementary Materials), highlighting the excellent sACE-inhibitory potential of AVQPCF, PIVCF, and CAIAW. The only peptides with IC_50_ values below the ones herein reported (3.69 µM or 2.45 µg/mL) were RYL (3.31 µM) [32] and IF (2.00 µM) [24], both identified in *Bombyx mori* hydrolysates (Table S2 Supplementary Materials). Other peptides exhibited values within the same range (<10 µM or <10 µg/mL) including AVFPSIVGR (6.64 µM) [26], IIAPPER (6.93 µg/mL) [33], KVEGDLK (3.67 µg/mL) [33], YETGNGIK (3.25 µg/mL) [33], AAAPVAVAK (8.31 µg/mL) [33], YDDGSYKPH (5.81 µg/mL) [33], and AGDDAPR (8.34 µg/mL) [33]. It is noteworthy that the peptide VF, which was isolated from *T. molitor* hydrolysates and demonstrated in vivo antihypertensive capacity, has a calculated IC_50_ of 230 µg/mL [34]. This value is threefold higher than the peptide evaluated in this study, with the highest IC_50_ (76.3 µg/mL for QIVW).

Further comparative analysis can also be conducted with the most extensively studied ACE-inhibitory peptides, IPP and VPP, which are found in fermented milks and have IC_50_ values for sACE of 5.00 and 9.00 µM, respectively [35], and are comparable to those found for AVQPCF, PIVCF, and CAIAW [36].

### 3.2. Peptide Detection in the GI Digesta by LC-MS/MS

**Figure 1:**
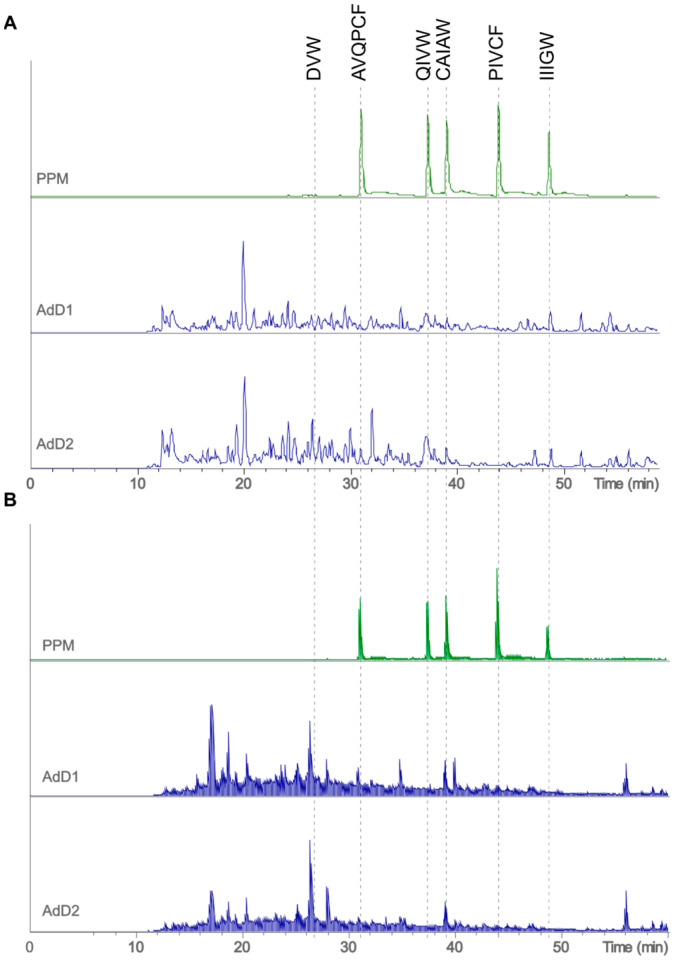
(**A**) Base peak chromatogram (BPC) and (**B**) total ion current (TIC) comparisons, where the top panel of each group (**A**,**B**) presents the chromatogram of the pure peptide mixture (PPM) (DVW (250 fmol), AVQPCF, QIVW, CAIAW, PIVCF, and IIIGW (50 fmol)), and the following panels show the chromatogram of *Acheta domesticus* digests (350 ng) 1 (AdD1) and 2 (AdD2), respectively (in blue).

To ascertain whether these peptides originated from in vitro GI digestion as indicated by the in silico results, a targeted approach was employed to determine their presence in two independent digestions of *A. domesticus* using LC-MS/MS, which helped to elucidate the structural identification. To confirm the presence of the peptides in the digested *A. domesticus* samples, the spectra of pure peptides were used for comparative purposes. The base peak chromatogram (BPC), together with the total ion current (TIC) of the pure peptide mixture (PPM) and *A. domesticus* digests 1 and 2 (AdD1 and AdD2), are presented in Figure 1.

**Figure 2:**
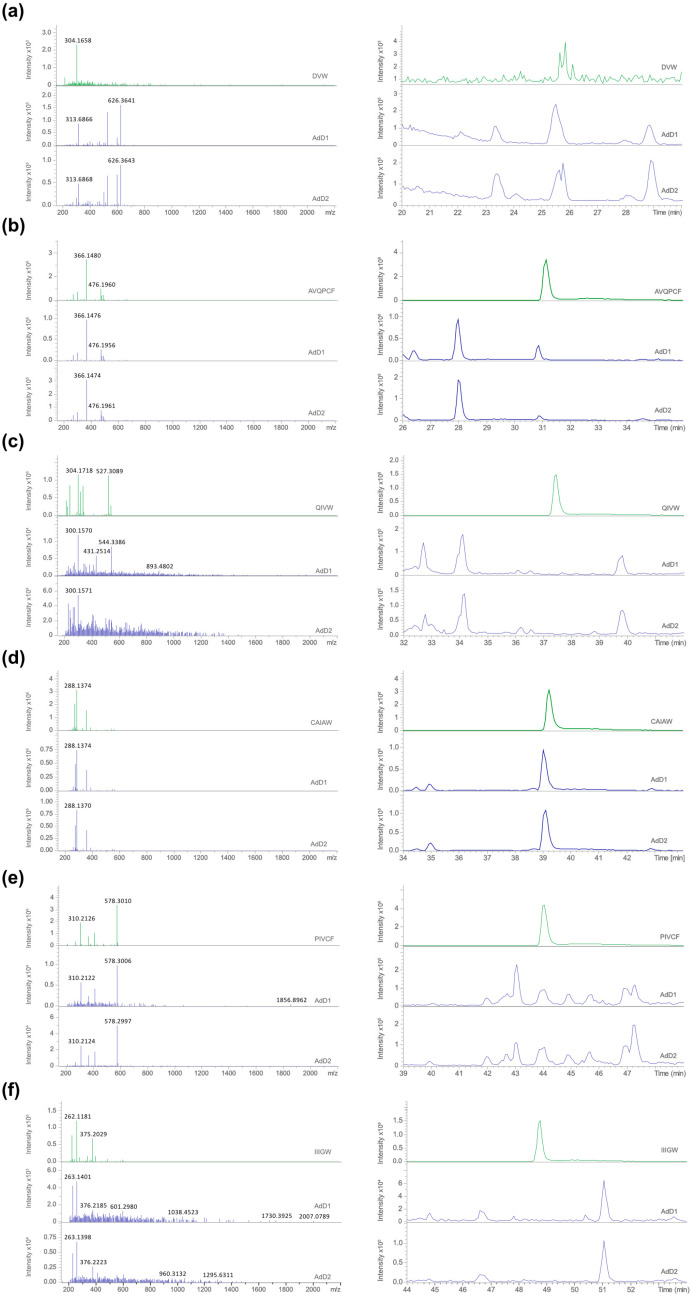
Base peak chromatogram (BPC) comparison (**right column**) and MS/MS spectra comparison (**left column**). In both cases, the top panel illustrates the BPC (**right column**) or MS/MS (**left column**) for the pure peptides (in green), and in the following panels, the *A. domesticus* digests 1 (AdD1) and 2 (AdD2), respectively (in blue). Each analyzed peptide corresponds to each figure item (**a**–**f**). For the BPC, all chromatograms (pure peptides and digests) were constructed from the specific *m*/*z* value identified in the MS/MS spectra for each analyzed peptide: (**a**) DVW (250 fmol) *m*/*z* of 419.1925; (**b**) AVQPCF (50 fmol) *m*/*z* of 664.3125; (**c**) QIVW (50 fmol) *m*/*z* of 545.3083; (**d**) CAIAW (50 fmol) *m*/*z* of 563.2647; (**e**) PIVCF (50 fmol) *m*/*z* of 578.3010; (**f**) IIIGW (50 fmol) *m*/*z* of 601.3708. For the MS/MS, the pure peptide spectra were obtained with the same *m*/*z* presented for the BPC, while the digests (350 ng) were performed with the following *m*/*z* values: (**a**) AdD1 *m*/*z* of 419.1934 and AdD2 *m*/*z* of 419.1923; (**b**) AdD1 *m*/*z* of 664.3127 and AdD2 *m*/*z* of 634.3129; (**c**) AdD1 *m*/*z* of 545.3069 and AdD2 *m*/*z* of 545.3045; (**d**) AdD1 *m*/*z* of 563.2630 and AdD2 *m*/*z* of 563.2645; (**e**) AdD1 *m*/*z* of 578.3006 and AdD2 *m*/*z* of 578.2997; (**f**) AdD1 *m*/*z* of 601.2989 and AdD2 *m*/*z* of 601.2625.

It must be noted that despite using a higher quantity of DVW (250 fmol vs. 50 fmol for the other peptides), the resolution hampered its identification, a fact that could be explained by the small size of the pure peptide. Figure 2 presents additional features; BPC is depicted in the left column, representing the selected [M + H]^+^, while the MS/MS spectra for these selected [M + H]^+^ ions are illustrated in the right column. The MS/MS method allowed us to obtain the *m*/*z* values of the fragmented ions. The *m*/*z* values of the original molecule and the newly formed fragments (product ions) are visualized in the mass spectrum of the metabolite. A mass spectrum of fragments is characteristic of a metabolite, and to some degree can be used to identify the original precursor by spectra comparison.

In Table 2, the “confirmed by MS/MS” column indicates whether the fragmentation pattern (spectrum) observed for a particular ion in the sample matched that of a known pure peptide. This means that the fragmentation pattern of the ion from the sample was the same or very similar to that of a known pure peptide (as seen in Figure 2). This provides strong evidence that the ion in the sample is the same as the standard. Therefore, the results clearly show that the three peptides, AVQPCF, CAIAW, and PIVCF, with excellent sACE-inhibitory potential, were present in the two independent digestions of *A. domesticus* (AdD1 and AdD2).

Additionally, the results also revealed the peptide relative quantities. CAIAW exhibited the highest content (approximately 4 µM), being about three times more abundant than AVQPCF and 15 times more than PIVCF. Since the IC_50_ values obtained for the ACE-inhibitory capacity of these peptides did not show a statistically significant difference (Table 1), it can be concluded that, despite their different concentrations in the digests, their individual contributions did not affect the overall ACE-inhibitory capacity.

### 3.3. Assessment of sACE-Inhibitory Capacity of A. domesticus GI Digesta

The results showed that the simulated GI digestion of *A. domesticus* yielded a digesta with an ACE-inhibitory capacity, having an IC_50_ value of 77.1 ± 11.8 µg protein/mL extract. Data indicate that the inhibition value observed for the *A. domesticus* digesta was comparable with those reported in the literature for other insect species (Table S2, Supplementary Materials). Distinct values of IC_50_ were described by different authors for several insect species, namely 30 µg/mL (calculated from the % values of inhibition) for *Protaetia brevitarsis* larvae hydrolysates [37], 28.3 μg/mL for the *B. mori* protein fraction [38], 230 µg/mL for the *T. molitor* protein fraction [39], and 1.9 µg/mL for a protein hydrolysate of *Gryllodes sigillatu* [40]. Additionally, de Matos et al. [41] reported an inhibition between 37.7 and 50.8% for *Gryllus assimilis* hydrolysates.

Although some authors reported higher sACE-inhibitory potential than that observed in this study, it must be noted that most of these values pertain to protein concentrates and/or fractions that have undergone some pre-treatments. This includes enzymatic hydrolysis with the objective of increasing the number of small peptides and separation techniques (charge, size) to concentrate the most promising ones. In this study, the sACE-inhibitory capacity of *A. domesticus* digesta was evaluated using whole insects, without any preliminary treatment or protein concentration, to ascertain its potential bioactivity in its minimally processed form, thus mimicking how they are commercialized within the EU.

The same comparison can be performed between the sACE-inhibitory capacity of *A. domesticus* digesta (IC_50_ of 77.1 µg/mL), with the most common protein sources broadly studied in the last decades such as milk (IC_50_ 323.1 µg/mL) [42], whey (IC_50_ 105.4 µg/mL) [27], egg (IC_50_ 221–272 µg/mL) [43], soybean (IC_50_ 340 µg/mL) [44], wheat germ (IC_50_ 452 µg/mL) [45], meat (e.g., beef rump (IC_50_ 116–259 µg/mL)) [46], fish (e.g., dried bonito (IC_50_ 22 µg/mL)) [47], and sardine (IC_50_ 62,400 µg/mL) [48]. In line with the previous comparison and even though some of the hydrolysates retrieved from the literature were submitted to different pre-treatment/concentration techniques, the IC_50_ values obtained for the *A. domesticus* digesta clearly stand out as a promising source of sACE-inhibitory peptides.

## 4. Conclusions

In this study, the sACE-inhibitory capacity of six out of seven peptides, previously identified by the in silico GI digestion of *A. domesticus* proteins, was confirmed, exhibiting IC_50_ values ranging between 3.69 and 195.5 µM. AVQPCF, PIVCF, and CAIAW stood out as the peptides with the lowest IC_50_ values (3.69 ± 0.25, 4.63 ± 0.16, and 6.55 ± 0.52 µM, respectively), being identified by LC-MS/MS in the *A. domesticus* digesta. Thus, the excellent results obtained validated the in silico protocol as a valuable tool for prospecting peptides with sACE-inhibitory capacity derived from known protein sequences. Moreover, the sACE-inhibitory capacity of *A. domesticus* whole insects subjected to GI digestion, without any preliminary treatment or protein concentration, was evidenced for the first time. This digesta showed a sACE-inhibitory capacity of 77.1 ± 11.8 µg protein/mL extract (IC_50_). The results clearly demonstrated that *A. domesticus* is not only a nutritionally valuable protein source, competing with other well-established sources commercially available, but, according to the IC_50_ values, also suggests that its consumption can mediate important beneficial health effects. Additional research will be carried out to confirm the in vivo antihypertensive properties of the most promising peptides.

## Figures and Tables

**Table 1:** Sequences of peptides and corresponding in vitro sACE-inhibitory capacity, measured by IC_50_, of the corresponding synthetic peptides. Identification of the protein source via sequence interpretation and database searching.

Amino Acid Sequence	Source Protein	ACE-Inhibitory Capacity Expressed as IC_50_ *	
		(µg/mL)	(µM)
DVW	Acyl-CoA Delta12-desaturase	81.8 ± 19.8 ^a^	195.5 ± 47.4 ^A^
AVQPCF	Diuretic hormone receptor	2.45 ± 0.17 ^b^	3.69 ± 0.25 ^B^
CAIAW		3.69 ± 0.29 ^b^	6.55 ± 0.52 ^B^
IIIGW		32.8 ± 7.5 ^c^	54.5 ± 12.6 ^C^
PIVCF	Acyl-CoA Delta-9 desaturase	2.68 ± 0.10 ^b^	4.63 ± 0.16 ^B^
QIVW		76.3 ± 5.6 ^a^	140.1 ± 10.4 ^A^

* Concentration of peptide needed to inhibit 50% of the original sACE activity (mean ± SD, *n* = 3); ^a–c^ and ^A–C^ Means that do not share the same letter are significantly different from each other at the 0.05 significance level by ANOVA analysis and Tukey’s post hoc test.

**Table 2:** Overview of the detected ions for the pure peptide mixture (PPM) and *A. domesticus* digests 1 and 2 (AdD1 and AdD2). The column [M + H]^+^_Calc_. represents the calculated monoisotopic expressed as (*m*/*z*) for each single charged peptide ion, the retention time (RT) in minutes, accompanied by the standard deviation based on six injections for SPM, and two technical replicates for AdD1 and AdD2. The ‘Confirmed by the MS/MS’ column indicates whether the MS/MS spectrum of the sample aligned with the MS/MS spectrum of the pure peptide. Quantitative results of the total peptides of AdD and AdD2 are presented as the average values with the standard deviation (SD) (*n* = 4).

	Peptide Content	Retention Time (Mean ± SD) (min)	Ion for MS/MS [M + H]^+^_Calc._	Observed Mass (Da)	Amino Acid Sequence	Confirmed by MS/MS
**Pure peptide mixture (PPM)**	250 nM for DVW 50 nM each for AVQPCF, QIVW, CAIAW, PIVCF, and IIIGW	26.2 ± 0.9	419.1925	419.1925	DVW	
		31.2 ± 0.4	664.3123	664.3123	AVQPCF	
		37.4 ± 0.4	545.3082	545.3082	QIVW	
		39.2 ± 0.4	563.2646	563.2646	CAIAW	
		44.1 ± 0.3	578.3007	578.3007	PIVCF	
		48.8 ± 0.4	601.3708	601.3708	IIIGW	
***Acheta domesticus* digesta 1 (AdD1)**	90.2 ± 0.5 mg/mL	25.6 ± 0.4	419.1925	419.1934	DVW	No
		30.9 ± 0.2	664.3123	664.3127	AVQPCF	Yes
		37.7 ± 0.4	545.3082	545.3069	QIVW	No
		39.4 ± 0.4	563.2646	563.2630	CAIAW	Yes
		44.0 ± 0.4	578.3007	578.3006	PIVCF	Yes
		-	601.3708	n. d.	IIIGW	No
***Acheta domesticus* digesta 2 (AdD2)**	71.8 ± 0.7 mg/mL	25.6 ± 0.5	419.1925	419.1923	DVW	No
		30.9 ± 0.1	664.3123	664.3129	AVQPCF	Yes
		37.8 ± 0.4	545.3082	545.3045	QIVW	No
		39.1 ± 0.4	563.2646	563.2645	CAIAW	Yes
		44.0 ± 0.4	578.3007	578.2997	PIVCF	Yes
		-	601.3708	n. d.	IIIGW	No

n. d.—Not detected.

## Data Availability

The original contributions presented in the study are included in the article/Supplementary Materials, further inquiries can be directed to the corresponding authors.

## Supplementary Materials

The following supporting information can be downloaded at: https://www.mdpi.com/article/10.3390/foods13213462/s1, Table S1: Composition of the simulated fluids used in the simulated GI digestion; Table S2: Summary of the main characteristics of the bioactive peptides identified in insect hydrolysates (source, sequence, IC_50_, % of inhibition, in vivo activity) and the methodology used for its production, isolation, and identification. References [49,50,51,52,53,54,55,56,57,58] are cited in the Supplementary Materials.
