# Supplementary material for: Uncovering the Potential Somatic Angiotensin-Converting Enzyme (sACE) Inhibitory Capacity of Peptides from Acheta domesticus: Insights from In Vitro Gastrointestinal Digestion
Source: Foods. 2024 Oct 29;13(21):3462. doi: 10.3390/foods13213462 (PMC11544891; doi:10.3390/foods13213462)
Supplement: Supplementary file 1 [file foods-13-03462-s001.zip › foods-3248590-supplementary.pdf]

Table S1. Composition of the simulated fluids used in the simulated gastrointestinal (GI) digestion.

	Simulated Salivary Fluid (SSF)	Simulated Gastric Fluid (SGF)	Simulated Intestinal Fluid (SIF)
NaCl (mM)	-	47.2	38.4
KCl (mM)	15.1	6.9	6.8
$NaHCO_3$ (mM)	13.6	25	85
KH_2PO_4 (mM)	3.7	0.9	0.8
$MgCl_2$ (mM)	0.15	0.12	0.33
$(NH4)_2CO_3$ (mM)	0.06	0.5	-
$CaCl_2(H_2O)_2$ (mM)	1.5	0.15	0.6
HCl (mM)	1.1	15.6	8.4
pH	7	3	7

Table S2. Summary of the main characteristics of the bioactive peptides identified in insect hydrolysates (source, sequence, IC_{50}, % of inhibition, *in vivo* activity) and the methodology used for its production, isolation, and identification.

Species	Sample	Sample Treatment	Type of GI Digestion	Analytical Method	Peptide Sequence	*In Vitro* (IC_{50} or % Inhibition)/ *in Vivo* Outputs	Reference
Protaetia brevitarsis (white-spotted flower chafer)	Larva	enzymatic proteolysis: flavourzyme	–	UHPLC-ESI-MS/MS	Total hydrolysate	87.09 ± 2.30 %, 63.62 ± 0.00 %, and 29.98 ± 8.52 % at 150, 30, and 15 μg/mL	[1]
					SY	–	
					PF	39.37 % at 150 μg/mL	
					YPY	–	
					WI	99.75 %, 98.98 %, and 95.67 % at 150, 30, and 15 μg/mL	
Apis mellifera (honeybee)	Three-day-old larva	–	*in vitro* (pepsin, trypsin and α-chymotrypsin (a))	UPLC/Q-TOF-MS	LLKPY	54.9 μM	[2]
	Freeze-dried granules of honeybee pupae of drones	enzymatic proteolysis: neutrase	*in silico*: Peptide Cutter (pepsin, trypsin, chymotrypsin (b))	LC–MS/MS	AVFPSIVGR	6.64 μM	[3]
					PGKVHIT	223.869 μM	
			in silico: Peptide Cutter (pepsin, chymotrypsin (b))		PPVLVFV	47.786 μM	
Bombyx mori (silkworm)	defatted pupae	enzymatic proteolysis: alcalase	–	Edman degradation	VEIS	–	[4]
			–	–	Protein fraction	28.3 μg/mL	
	Pupae powder	–	*in vitro* (pepsin, trypsin, and α-chymotrypsin (a))	IT-MS/MS	ASL	102.15 μM	[5]
	Pupae	enzymatic proteolysis: neutral protease	*in vitro* (pepsin, pancreatin (a))	MALDI-TOF/TOF-MS	RYL	3.31 ± 0.11 μM	[6]
	Pupae	enzymatic proteolysis: neutral alcalase	*in vitro* (pepsin, pancreatin (b))	MALDI-TOF/TOF MS	GAMVVH	19.39 ±0.21 μM	[7]
	Silkworm pupa powder	ultrasonic pre-treatment; enzymatic proteolysis (alcalase)	*in vitro* (pepsin, trypsin, and α-chymotrypsin (b))	IT-MS/MS	KHV	12.82 μM	[8]
	Defatted dry pupae	enzymatic proteolysis (acidic protease)	–	LC–MS/MS	APPPKK	47 μg/mL	[9]
	Pupae	enzymatic proteolysis (neutral protease)	*in vitro* (pepsin, pancreatin (b))	MALDI-TOF/TOF MS	GNPWM	21.70 μM	[10]
	Pupae protein sequences (OMICS Scientific Database)	–	*in silico*: PeptideCutter (all available enzymes and chemicals provided by the software (a))	–	IF	2.00±0.12 μM	[11]
					GD	12500.00±0.2 μM	
					DA	223.00±0.25 μM	
					TE	673.00±0.14 μM	
					TA	594.00±0.018 μM	
					ES	7937.00±0.16 μM	
					SS	541.00±0.21 μM	
					ST	1052.00±0.34 μM	
					SD	12113.00±0.28 μM	

	Actin sequence (ACT1_BOMMO)	–	*in silico*: PeptideCutter (pepsin, trypsin and α-chymotrypsin (a))	–	QD<br>QE<br>EG<br>DL<br>GM<br>QK	4019.00±0.16 µM<br>1410.00±0.27 µM<br>10000 µM*<br>2000 µM*<br>1400 µM*<br>885 µM*	[12]
Gryllodes sigillatu (tropical banded cricket)	Whole insect	enzymatic proteolysis (alcalase)	*in vitro* (pepsin, pancreatin (a))	LC–MS/MS	YKPRP<br>PHGAP<br>VGPPQ	1.922 µg/mL	[13]
		Raw<br>Boiled<br>Baked<br>Protein	*in vitro* (pepsin, pancreatin (a))	LC-MS	IIAPPER<br>LAPSTIK<br>VAPEEHPV<br>KVEGDLK	6.93 ± 0.05 µg/mL<br>11.14 ± 0.08 µg/mL<br>18.85 ± 0.05 µg/mL<br>3.67 ± 0.02 µg/mL	[14]
Gryllus assimilis (black cricket)	Whole insect	enzymatic proteolysis (Flavourzyme and Neutrase)<br>enzymatic proteolysis (Flavourzyme and Alcalase)	–	nanoLC-ESI-MS/MS	AFLL<br>LPLP<br>DM(+15.99)EKI<br>WH<br>VFPSIVGRPR<br>ASTSLEKSY<br>AGDDAPR<br>NILFSGTNVA<br>AGKAR<br>NPEGLLTGRPR<br>RYDPNRVF	37.66 % ± 5.64<br>50.84 %± 3.38	[15]
Musca domestica (housefly)	Sequences of the eight major proteins of larvae	–	*in silico*: BIOPEP-UWM (pepsin, trypsin, and chymotrypsin A (a))	–	KPYDLGGRMF<br>YPLDL<br>WGPTKPP<br>AF<br>GW<br>GY<br>PH<br>VF	–	[16]
Oecophylla smaragdina (weaver ant)	Larvae and pupae	–	*in vitro* (pepsin,trypsin (a))	LC-MS/MS	FFGT<br>LSRVP	19.45 ± 1.70 µM<br>52.73 ± 4.04 µM	[17]
Schistocerca gregaria (desert locust)	Full adult insect	Raw<br>Boiled<br>Baked<br>Protein	*in vitro* (pepsin, pancreatin (a))	LC-MS	GKDAVIV<br>AIGVGAIER<br>FDPFPK<br>YETGNGIK	12.82 ± 0.02 µg/mL<br>14.43 ± 0.02 µg/mL<br>79.25 ± 0.04 µg/mL<br>3.25 ± 0.09 µg/mL	[14]
Spodoptera littoralis (cotton leafworm)	Larvae	–	*in vitro* (pepsin, trypsin and α-chymotrypsin (a))	automated Edman	AVF	2123 µM	[18]
	Synthesized peptides AVF and VF	–	*in vitro* (pepsin, trypsin and α-chymotrypsin (b))	–	AVF<br><br>VF	1374-1430 µM<br>no inhibitory activity in thoracic aortic rings from male Wistar rats<br>anti-hypertensive activity in SHR<br>120-144 µM<br>inhibitory activity in thoracic aortic rings from male Wistar rats<br>anti-hypertensive activity (higher than AVF) in SHR	[19]
Tenebrio molitor (mealworm)	Larva flour	enzymatic proteolysis: alcalase	–	UPLC-MS/MS<br>–	YAN<br>protein fraction	17 µg/mL<br>230 µg/mL<br>anti-hypertensive activity (significant reduction of systolic blood pressure) in SHR	[20]
	Larvae	–	*in vitro* (pepsin, trypsin and α-chymotrypsin (a))	LC-HRMS/MS	QGLGY<br>NIKY<br>HILG	264 µM<br>52.2 µM<br>2820 µM	[21]

					YAN (target approach)	172.1 μM	
	Larvae	Raw<br>Boiled<br>Baked<br>protein concentrate	*in vitro* (pepsin, pancreatin (a))	LC-MS	NYVADGLG<br>AAAPVAVAK<br>YDDGSYKPH<br>AGDDAPR	12.09 ± 0.08 μg/mL<br>8.31 ± 0.02 μg/mL<br>5.81 ± 0.02 μg/mL<br>8.34 ± 0.02 μg/mL	[14]

MD—molecular docking; SHR—spontaneously hypertensive rats; (a) enzymes used to obtain the peptides; (b) enzymes used to evaluate peptide stability. * obtained with sACE inhibitory peptide database—Agrotechnology and Food Innovations in Wageningen, the Netherlands.

References

1. Lee, J.H.; Kim, T.-K.; Yong, H.I.; Cha, J.Y.; Song, K.-M.; Lee, H.G.; Je, J.-G.; Kang, M.-C.; Choi, Y.-S. Peptides inhibiting angiotensin-I-converting enzyme: Isolation from flavourzyme hydrolysate of Protaetia brevitarsis larva protein and identification. *Food Chemistry* **2023**, *399*. https://doi.org/10.1016/j.foodchem.2022.133897.
2. Xu, X.; Gao, Y.X. Purification and identification of angiotensin I-converting enzyme-inhibitory peptides from apalbumin 2 during simulated gastrointestinal digestion. *Journal of the Science of Food and Agriculture* **2015**, *95*, 906-914. https://doi.org/10.1002/jsfa.6755.
3. Yang, X.; Chen, K.; Liu, H.; Zhang, Y.; Luo, Y. Purification and identification of peptides with high angiotensin-I converting enzyme (ACE) inhibitory activity from honeybee pupae (Apis mellifera) hydrolysates with in silico gastrointestinal digestion. *European Food Research and Technology* **2019**, *245*, 535-544. https://doi.org/10.1007/s00217-018-03223-7.
4. Li, X.; Li, Y.; Huang, X.; Zheng, J.; Zhang, F.; Kan, J. Identification and characterization of a novel angiotensin I-converting enzyme inhibitory peptide (ACEIP) from silkworm pupa. *Food Science and Biotechnology* **2014**, *23*, 1017-1023. https://doi.org/10.1007/s10068-014-0138-9.
5. Wu, Q.; Jia, J.; Yan, H.; Du, J.; Gui, Z. A novel angiotensin-I converting enzyme (ACE) inhibitory peptide from gastrointestinal protease hydrolysate of silkworm pupa (Bombyx mori) protein: Biochemical characterization and molecular docking study. *Peptides* **2015**, *68*, 17-24. https://doi.org/10.1016/j.peptides.2014.07.026.
6. Liu, L.; Wei, Y.; Chang, Q.; Sun, H.; Chai, K.; Huang, Z.; Zhao, Z.; Zhao, Z. Ultrafast Screening of a Novel, Moderately Hydrophilic Angiotensin-Converting-Enzyme-Inhibitory Peptide, RYL, from Silkworm Pupa Using an Fe-Doped-Silkworm-Excrement-Derived Biocarbon: Waste Conversion by Waste. *Journal of Agricultural and Food Chemistry* **2017**, *65*, 11202-11211. https://doi.org/10.1021/acs.jafc.7b04442.
7. Tao, M.; Sun, H.; Liu, L.; Luo, X.; Lin, G.; Li, R.; Zhao, Z.; Zhao, Z. Graphitized Porous Carbon for Rapid Screening of Angiotensin-Converting Enzyme Inhibitory Peptide GAMVVH from Silkworm Pupa Protein and Molecular Insight into Inhibition Mechanism. *Journal of Agricultural and Food Chemistry* **2017**, *65*, 8626-8633. https://doi.org/10.1021/acs.jafc.7b03195.
8. Jia, J.; Wu, Q.; Yan, H.; Gui, Z. Purification and molecular docking study of a novel angiotensin-I converting enzyme (ACE) inhibitory peptide from alcalase hydrolysate of ultrasonic-pretreated silkworm pupa (Bombyx mori) protein. *Process Biochemistry* **2015**, *50*, 876-883. https://doi.org/10.1016/j.procbio.2014.12.030.
9. Wang, W.; Wang, N.; Zhou, Y.; Zhang, Y.; Xu, L.; Xu, J.; Feng, F.; He, G. Isolation of a novel peptide from silkworm pupae protein components and interaction characteristics to angiotensin I-converting enzyme. *European Food Research and Technology* **2010**, *232*, 29-38. https://doi.org/10.1007/s00217-010-1358-8.
10. Tao, M.; Wang, C.; Liao, D.; Liu, H.; Zhao, Z.; Zhao, Z. Purification, modification and inhibition mechanism of angiotensin I-converting enzyme inhibitory peptide from silkworm pupa (Bombyx mori) protein hydrolysate. *Process Biochemistry* **2017**, *54*, 172-179. https://doi.org/10.1016/j.procbio.2016.12.022.
11. Wang, W.; Zhang, Y.; Wang, N.; Zhu, Z. Molecular mechanisms of several novel dipeptides with angiotensin-iconverting enzyme inhibitory activity from in-silico screening of silkworm pupae protein. *Curr. Pharm. Biotechnol.* **2014**, *15*, 691-699. https://doi.org/10.2174/1389201015081409301533336.
12. Vercruysse, L.; Smagghe, G.; van der Bent, A.; van Amerongen, A.; Ongenaert, M.; Van Camp, J. Critical evaluation of the use of bioinformatics as a theoretical tool to find high-potential sources of ACE inhibitory peptides. *Peptides* **2009**, *30*, 575-582. https://doi.org/10.1016/j.peptides.2008.06.027.
13. Hall, F.; Reddivari, L.; Liceaga, A.M. Identification and Characterization of Edible Cricket Peptides on Hypertensive and Glycemic In Vitro Inhibition and Their Anti-Inflammatory Activity on RAW 264.7 Macrophage Cells. *Nutrients* **2020**, *12*. https://doi.org/10.3390/nu12113588.
14. Zielińska, E.; Karaś, M.; Baraniak, B.; Jakubczyk, A. Evaluation of ACE, α-glucosidase, and lipase inhibitory activities of peptides obtained by in vitro digestion of selected species of edible insects. *European Food Research and Technology* **2020**, *246*, 1361-1369. https://doi.org/10.1007/s00217-020-03495-y.
15. de Matos, F.M.; de Lacerda, J.T.J.G.; Zanetti, G.; de Castro, R.J.S. Production of black cricket protein hydrolysates with α-amylase, α-glucosidase and angiotensin I-converting enzyme inhibitory activities using a mixture of proteases. *Biocatal. Agric. Biotechnol.* **2022**, *39*. https://doi.org/10.1016/j.bcab.2022.102276.
16. Koh, J.A.; Ong, J.H.; Abd Manan, F.; Ee, K.Y.; Wong, F.C.; Chai, T.T. Discovery of Bifunctional Anti-DPP-IV and Anti-ACE Peptides from Housefly Larval Proteins After In silico Gastrointestinal Digestion. *Biointerface Research in Applied Chemistry* **2022**, *12*, 4929-4944. https://doi.org/10.33263/briac124.49294944.

17. Pattarayingsakul, W.; Nilavongse, A.; Reamtong, O.; Chittavanich, P.; Mungsantisuk, I.; Mathong, Y.; Prasitwuttisak, W.; Panbangred, W. Angiotensin-converting enzyme inhibitory and antioxidant peptides from digestion of larvae and pupae of Asian weaver ant, Oecophylla smaragdina, Fabricius. *Journal of the Science of Food and Agriculture* **2017**, *97*, 3133-3140. https://doi.org/10.1002/jsfa.8155.
18. Vercruysse, L.; Smagghe, G.; Matsui, T.; Van Camp, J. Purification and identification of an angiotensin I converting enzyme (ACE) inhibitory peptide from the gastrointestinal hydrolysate of the cotton leafworm, Spodoptera littoralis. *Process Biochemistry* **2008**, *43*, 900-904. https://doi.org/10.1016/j.procbio.2008.04.014.
19. Vercruysse, L.; Van Camp, J.; Morel, N.; Rouge, P.; Herregods, G.; Smagghe, G. Ala-Val-Phe and Val-Phe: ACE inhibitory peptides derived from insect protein with antihypertensive activity in spontaneously hypertensive rats. *Peptides* **2010**, *31*, 482-488. https://doi.org/10.1016/j.peptides.2009.05.029.
20. Dai, C.; Ma, H.; Luo, L.; Yin, X. Angiotensin I-converting enzyme (ACE) inhibitory peptide derived from Tenebrio molitor (L.) larva protein hydrolysate. *European Food Research and Technology* **2013**, *236*, 681-689. https://doi.org/10.1007/s00217-013-1923-z.
21. Brai, A.; Immacolata Trivisani, C.; Vagaggini, C.; Stella, R.; Angeletti, R.; Iovenitti, G.; Francardi, V.; Dreassi, E. Proteins from Tenebrio molitor: An interesting functional ingredient and a source of ACE inhibitory peptides. *Food Chemistry* **2022**, *393*. https://doi.org/10.1016/j.foodchem.2022.133409.

Disclaimer/Publisher's Note: The statements, opinions and data contained in all publications are solely those of the individual author(s) and contributor(s) and not of MDPI and/or the editor(s). MDPI and/or the editor(s) disclaim responsibility for any injury to people or property resulting from any ideas, methods, instructions or products referred to in the content.
